# Percutaneous cannulation is associated with lower rate of severe neurological complication in femoro-femoral ECPR: results from the Extracorporeal Life Support Organization Registry

**DOI:** 10.1186/s13613-023-01174-1

**Published:** 2023-08-30

**Authors:** Liangshan Wang, Chenglong Li, Xin Hao, Peter Rycus, Joseph E. Tonna, Peta Alexander, Eddy Fan, Hong Wang, Feng Yang, Xiaotong Hou

**Affiliations:** 1 Center for Cardiac Intensive Care, Beijing Anzhen Hospital, Capital Medical University, Beijing, People’s Republic of China; 2 Extracorporeal Life Support Organization (ELSO), University of Michigan, Ann Arbor, MI USA; 3 Division of Emergency Medicine, Division of Cardiothoracic Surgery, Department of Surgery, University of Utah Health, Salt Lake City, UT USA; 4 Department of Cardiology, Department of Pediatrics, Boston Children’s Hospital, Harvard Medical School, Boston, MA USA; 5 Interdepartmental Division of Critical Care Medicine, University of Toronto, Toronto, ON Canada

**Keywords:** Extracorporeal cardiopulmonary resuscitation, Cardiac arrest, Percutaneous cannulation, Surgical cannulation, Severe neurological complication

## Abstract

**Background:**

Percutaneous cannulation is now accepted as the first-line strategy for extracorporeal cardiopulmonary resuscitation (ECPR) in adults. However, previous studies comparing percutaneous cannulation to surgical cannulation have been limited by small sample size and single-center settings. This study aimed to compare in-hospital outcomes in cardiac arrest (CA) patients who received femoro-femoral ECPR with percutaneous vs surgical cannulation.

**Methods:**

Adults with refractory CA treated with percutaneous (percutaneous group) or surgical (surgical group) femoro-femoral ECPR between January 2008 and December 2019 were extracted from the international Extracorporeal Life Support Organization registry. The primary outcome was severe neurological complication. Multivariable logistic regression analyses were performed to assess the association between percutaneous cannulation and in-hospital outcomes.

**Results:**

Among 3575 patients meeting study inclusion, 2749 (77%) underwent percutaneous cannulation. The proportion of patients undergoing percutaneous cannulation increased from 18% to 89% over the study period (*p* < 0.001 for trend). Severe neurological complication (13% vs 19%; *p* < 0.001) occurred less frequently in the percutaneous group compared to the surgical group. In adjusted analyses, percutaneous cannulation was independently associated with lower rate of severe neurological complication (odds ratio [OR] 0.62; 95% CI 0.46–0.83; *p* = 0.002), similar rates of in-hospital mortality (OR 0.93; 95% CI 0.73–1.17; *p* = 0.522), limb ischemia (OR 0.84; 95% CI 0.58–1.20; *p* = 0.341) and cannulation site bleeding (OR 0.90; 95% CI 0.66–1.22; *p* = 0.471). The comparison of outcomes provided similar results across different levels of center percutaneous experience or center ECPR volume.

**Conclusions:**

Among adults receiving ECPR, percutaneous cannulation was associated with probable lower rate of severe neurological complication, and similar rates of in-hospital mortality, limb ischemia and cannulation site bleeding.

**Supplementary Information:**

The online version contains supplementary material available at 10.1186/s13613-023-01174-1.

## Introduction

Extracorporeal cardiopulmonary resuscitation (ECPR), using veno-arterial extracorporeal membrane oxygenation (VA-ECMO) for circulatory and respiratory support, has been increasingly used worldwide as a rescue strategy in patients with refractory cardiac arrest (CA) [1–8]. Recent investigations have shown that ECPR is associated with improved survival and neurologic outcomes in patients with in-hospital CA (IHCA) or out-of-hospital CA (OHCA) as compared to conventional cardiopulmonary resuscitation [9–14].

In adults, peripheral femoro-femoral cannulation is the most commonly adopted method for the initiation of ECPR, predominantly performed with percutaneous approach or surgical approach [15–17]. The surgical approach has been widely used for a long time, but this more invasive procedure may be associated with severe complications, such as hemorrhage, delayed wound healing, and infections [18, 19]. With the development of ultrasound guidance for vascular access and the notable advances in devices and techniques, percutaneous cannulation is increasingly performed by intensivists, cardiologists, interventional radiologists, surgeons, and emergency physicians, and is often preferred over surgical cannulation in most cases [20, 21]. Importantly, percutaneous canulation was shown to be related to fewer complications and shorter cannulation time, potentially improving outcomes in patients undergoing ECPR [22]. However, previous studies comparing percutaneous cannulation to surgical cannulation have been limited by small sample size and single-center settings or were conducted in populations with cardiogenic shock [23–25]. In addition, there is little information regarding trends in the utilization of vascular access techniques (percutaneous vs surgical cannulation) for ECPR over the past decades.

The aims of this study were to leverage the Extracorporeal Life Support Organization (ELSO) registry to: (1) evaluate temporal trends in the use of percutaneous vs surgical cannulation in ECPR; (2) compare in-hospital outcomes among CA patients who received femoro-femoral ECPR with percutaneous vs surgical cannulation; (3) quantify the association of percutaneous cannulation with in-hospital outcomes in prespecified subgroups.

## Methods

### Study design and population

The ELSO Registry is a voluntary international database that collects information on ECMO use in adults and children from more than 400 member centers worldwide. Records stored in the registry include patient demographics, diagnosis and procedure information, pre-ECMO conditions, ECMO technique, physiological and microbiological data, complications, and outcomes. Diagnosis and medical history are reported according to the International Classification of Diseases (ICD) 9th Edition and 10th Edition codes. We conducted a retrospective cohort study using the ELSO Registry. We included adult patients (≥ 18 years) who received femoro-femoral ECPR with percutaneous (percutaneous group) or surgical (surgical group) cannulation primarily for refractory CA from January 1, 2008, through December 31, 2019. We excluded patients with multiple VA-ECMO runs, central cannulation, and other nonfemoral arterial cannulation approaches. Additional exclusion criteria included missing data with regard to key variables: cannulation method and neurologic complications. This study was approved by the Research Ethics Board of the Beijing Anzhen Hospital (2021020X).

### Data collection

Two authors (F.Y and L.W) independently reviewed the deidentified data set from the ELSO Registry database and identified all patients meeting the inclusion criteria. Any discrepancies between the two reviewers were resolved by discussion. The following information was extracted for all included patients: demographic variables; comorbid conditions; CA characteristics; pre-ECMO support; pre-ECMO hemodynamic variables; pre-ECMO arterial blood gases (ABGs); post-ECMO ABGs; year of ECMO run; cannulation site; cannulation techniques; cannula type; VA-ECMO duration; and ECMO-associated mortality and morbidities, including neurologic complications, limb ischemia, cannulation site bleeding, infections, and need for renal replacement therapy (RRT). Comorbidities including hypertension, diabetes mellitus, coronary artery disease, chronic heart failure, chronic obstructive pulmonary disease, chronic kidney disease, and obesity (defined as a BMI ≥ 30 kg/m^2^). CA characteristics included cardiac origin of CA, OHCA, and witnessed initial shockable rhythm. Cardiac origin of CA was reported according to ICD-9/10 codes. Pre-ECMO support included renal replacement therapy (RRT), intra-aortic balloon pump (IABP), percutaneous ventricular assist device (PVAD), cardiopulmonary bypass use, norepinephrine and vasopressin use. The ELSO registry collects arterial blood gas values before initiation of ECMO and 24 h after the initiation of ECMO. The absolute change in PaCO2 upon ECMO initiation.

(ΔPaCO2) was calculated from the following formula: ΔPaCO2 = 24hPost_ECMO_PaCO2- Pre_ECMO_PaCO2. The relative change in PaCO2 (Rel∆CO2) was calculated from the following formula: Rel∆CO2 = ΔPaCO2/PreECMOPaCO2. Severe hyperoxemia was defined as PaO2 > 300 mmHg. Cannulation techniques were reported as percutaneous or surgical. Neurologic complications were reported as brain death, central nervous system (CNS) diffuse ischemia, ischemic stroke, hemorrhagic stroke, or seizure.

### Outcomes and definitions

The primary outcome was severe neurologic complication, defined as brain death or CNS diffuse ischemia (ELSO definitions in Additional file 1: Table S1). Secondary outcomes included in-hospital mortality, stroke, limb ischemia, cannulation site bleeding, systemic infection, respiratory tract infection, and need for RRT (ELSO definitions in Additional file 1: Table S1). In-hospital mortality was defined as death from any cause occurring in patients who were treated with ECPR for CA. Stroke included hemorrhagic stroke and ischemic stroke. Systemic infection was defined by a culture proven infection from blood during ECMO support and not believed to be pre-existing. Respiratory tract infection was defined by a culture proven infection from respiratory tract during ECMO support and not believed to be pre-existing.

### Statistical analysis

Continuous variables were expressed as median with interquartile range (IQR) and were compared with the Mann–Whitney *U* test. Categorical variables were expressed as counts and percentages and were compared with chi-square or Fisher’s exact tests. Patient characteristics and outcomes were compared between surgical and percutaneous groups. The percentage of patients undergoing percutaneous and surgical cannulation was plotted over time. Cochrane–Armitage test for trend was used to evaluate the use of percutaneous vs surgical cannulation over time.

To evaluate the association between percutaneous cannulation and outcomes, we performed multivariable logistic regressions by entering percutaneous cannulation (vs surgical cannulation) with available variables: age, weight, sex, race, comorbid conditions, cardiac origin, bilateral femoral cannula, distal limb cannula use, pre-ECMO PH, and VA-ECMO duration. Given a moderate level of missing data for pre-ECMO pH (32%) and pre-ECMO support (18%), we performed sensitivity analyses by repeating all logistic regression analyses by either omitting pH or including pre-ECMO support to assess their influence on outcomes. Sensitivity analyses with the year of ECPR entered in the models was also presented. Moreover, we explored if the association between percutaneous cannulation and neurological complications remained significant, once oxygenation variables (Rel∆CO2 and severe hyperoxemia) were entered in the multiple logistic regression model.

To assess for effect modification in the relationship between percutaneous cannulation and selected outcomes (severe neurologic complication, in-hospital mortality, limb ischemia, and cannulation site bleeding) in prespecified subgroups of interest defined according to age (< 50 years, 50–60 years, > 60 years), sex, obesity, white race, cardiac origin of CA, OHCA, witnessed CA, distal limb cannula use, pre-ECMO PH (< 7.2, > 7.2), and ECMO period (pre-2015, 2015–2019), multivariable logistic regression models as above including an interaction between percutaneous cannulation and the variable representing the subgroup were fitted in this study cohort.

We defined experience of percutaneous cannulation for each center as the total number of patients underdoing percutaneous ECPR at that center during study period. Centers with ≥ 5 cases of percutaneous ECPR was considered to be experienced in percutaneous cannulation (according to the median value of percutaneous ECPR volume across the included centers). We compared outcomes across different levels of center percutaneous experience with multivariable logistic regression models as above and tested for a statistical interaction between percutaneous cannulation and center experience of percutaneous cannulation. We also compared outcomes across different levels of center ECPR volume (two categories: < 30, and ≥ 30 cases; this categorization was adopted from a previous publication [26]) with multivariable logistic regression models as above and tested for a statistical interaction between percutaneous cannulation and center ECPR volume. P values less than 0.05 were considered to be statistically significant. All the analyses were performed with SPSS 25.0 (IBM Corp, Armonk, NY, USA) and R 4.0.3 (the R Foundation, https://www.r-project.org/).

## Results

### Patient characteristics

**Fig. 1:**
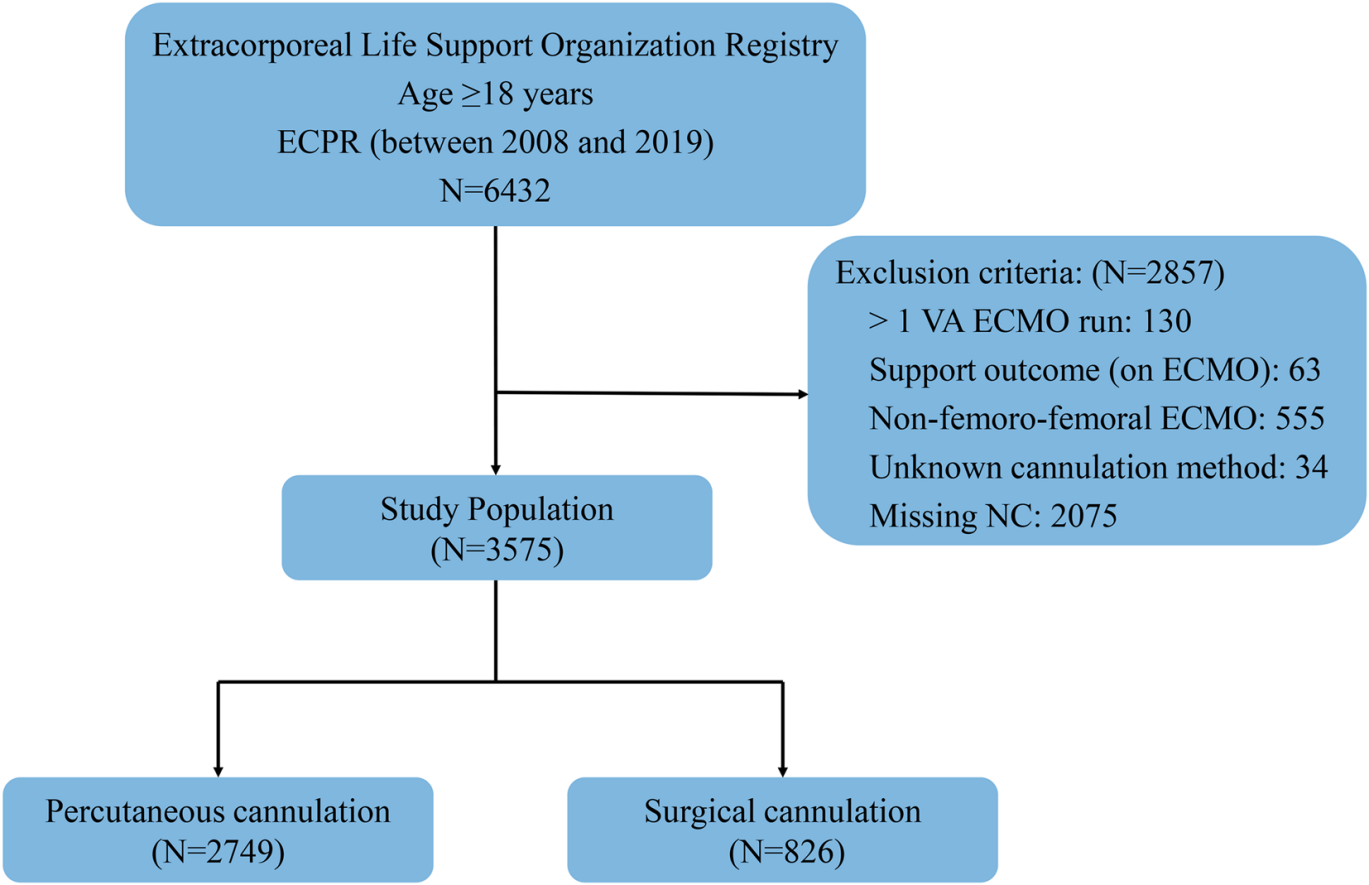
Flow diagram for selection of patients. *ECPR* extracorporeal cardiopulmonary resuscitation, *VA-ECMO* venoarterial extracorporeal membrane oxygenation, *NC* neurological complication

**Fig. 2:**
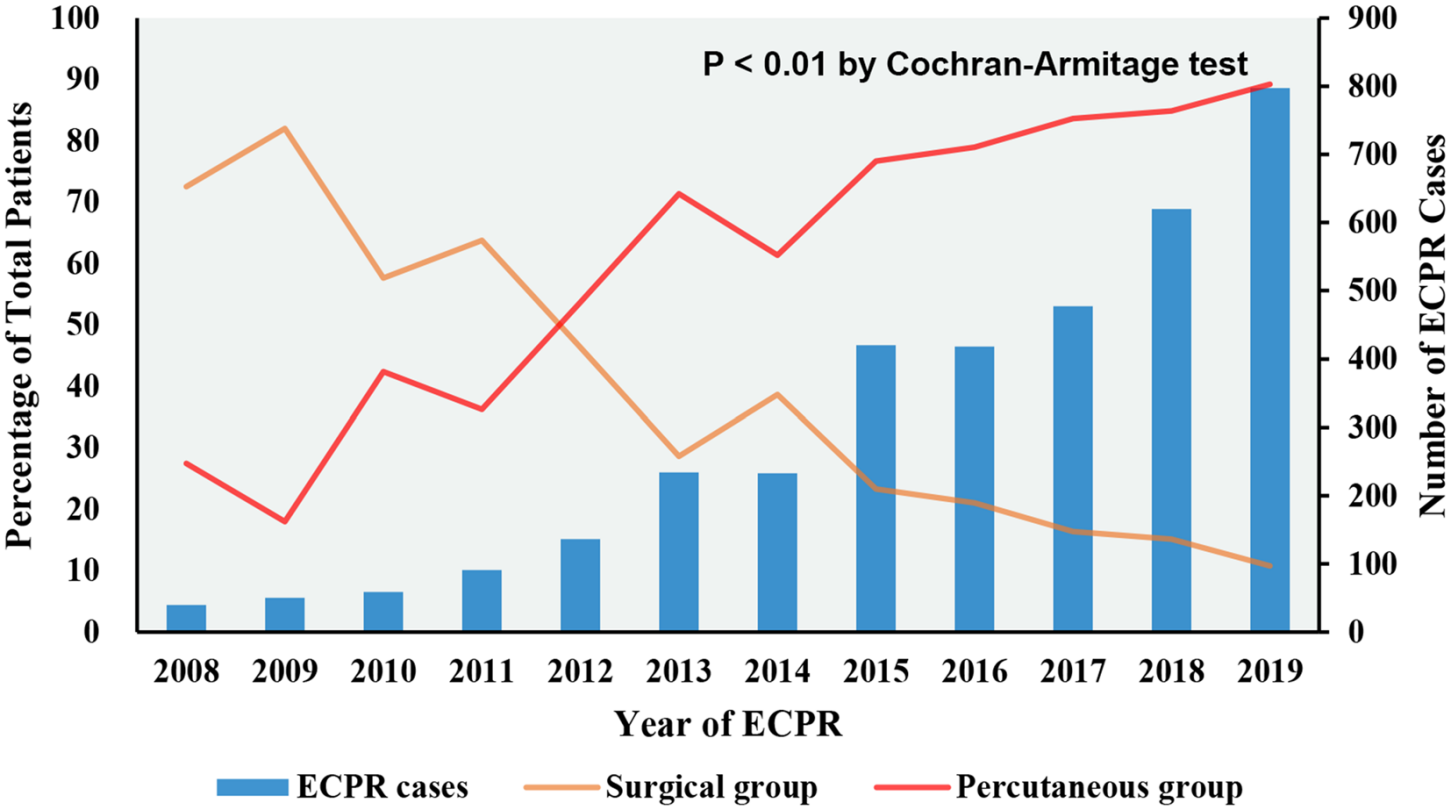
Percentage of all adult patients undergoing percutaneous and surgical femoro-femoral extracorporeal cardiopulmonary resuscitation (ECPR) from 2008 to 2019

Among 6432 adult patients receiving ECPR from January 1, 2008, to December 31, 2019, 3575 patients met the study’s inclusion criteria (Fig. 1). Percutaneous cannulation was used in 2749 patients (77%), whereas surgical cannulation was used in 826 cases (23%). The proportion of patients undergoing percutaneous cannulation increased over time from 18% to 89% over the study period (*p* value for trend < 0.001) and has been higher than that of patients undergoing surgical cannulation since 2012 (Fig. 2). The baseline characteristics of the patients are presented in Table 1. The median (IQR) age was 57 (45–66) years, the median (IQR) weight was 80 (70–98) kg, the median (IQR) BMI was 28 (24–33) kg/m^2^, and the majority of patients were white (61%) and were male (72%). The most common comorbid conditions were coronary artery disease (31%), obesity (23%), and hypertension (11%). CA of cardiac origin occurred in 2274 of 3575 (67%) patients, and OHCA occurred in 429 of 2017 (21%) patients. Three hundred and ninety-seven patients (14%) had an IABP placed before ECMO implantation, and 1228 (42%) patients required norepinephrine. The median (IQR) pH before cannulation was 7.14 (6.99–7.29). The median (IQR) ΔPaCO2 was −12(−31 to 0) mmHg, the median Rel∆CO2 was −26% (−48% to 0), and severe hyperoxemia was present in 658 of 2766 (24%) patients after the initiation of ECMO. Bilateral groin cannulation was performed in 1192 patients (33%), and distal limb cannula was used in 945 cases (26%). The median (IQR) time on VA-ECMO support was 3.0 (1.2–6.1) days. There was a significant difference between the percutaneous group and the surgical group in age, weight, BMI, race, hypertension, coronary artery disease, obesity, cardiac origin, baseline pH level, bilateral groin cannulation, and distal limb cannula placement (*p* < 0.05 for all).

**Table 1:** Baseline characteristics

Parameter [median (IQR)/*n* (%)]	Overall (*n* = 3575)	Percutaneous group (*n* = 2749)	Surgical group (*n* = 826)	*p* value
Age, years	57 (45–66)	57 (46–66)	56 (43–65)	0.045
Female^a^	989 (28)	751 (27)	238 (29)	0.451
Weight^a^ kg	80 (70–98)	84 (70–100)	75 (64–90)	< 0.001
BMI^a^ kg/m^2^	28 (24–33)	28 (24–33)	27 (24–31)	0.001
Race				
White	1926 (54)	1664 (61)	262 (32)	< 0.001
Asian	792 (22)	360 (13)	432 (52)	< 0.001
Black	337 (9)	285 (10)	52 (6)	< 0.001
Hispanic	135 (4)	110 (4)	25 (3)	0.076
Others	385 (11)	330 (12)	55 (7)	< 0.001
Comorbid conditions^a^				
Hypertension	373 (11)	306 (12)	67 (8)	0.003
Diabetes	280 (8)	226 (9)	54 (7)	0.057
Coronary artery disease	1069 (31)	849 (33)	220 (27)	0.002
Chronic heart failure	279 (8)	220 (8)	59 (7)	0.253
COPD	49 (1)	42 (2)	7 (1)	0.111
Chronic kidney disease	168 (5)	136 (5)	32 (4)	0.127
Obesity	781 (23)	679 (26)	102 (12)	< 0.001
CA characteristics^a^				
Cardiac origin	2274 (67)	1754 (68)	520 (64)	0.037
OHCA	429 (21)	323 (21)	106 (21)	0.717
Witnessed	1809 (89)	1341 (88)	468 (90)	0.283
Initial shockable rhythm	1088 (55)	822 (56)	266 (52)	0.111
Pre-ECMO support^a^				
Renal replacement therapy	110 (4)	91 (4)	19 (3)	0.070
Intra-aortic balloon	397 (14)	294 (13)	103 (14)	0.498
PVAD	105 (4)	96 (4)	9 (1)	< 0.001
Norepinephrine	1228 (42)	965 (44)	263 (36)	< 0.001
Vasopressin	253 (9)	232 (10)	21 (3)	< 0.001
Pre-ECMO ABG^a^				
PH	7.14 (6.99–7.29)	7.13 (6.98–7.27)	7.17 (7.00–7.33)	< 0.001
PaCO_2_, mmHg	49 (36–69)	50 (37–70)	47 (32–66)	< 0.001
PaO_2_, mmHg	73 (49–135)	74 (49–131)	73 (48–142)	0.816
HCO_3_, mmol/L	17.0 (13.0–21.6)	17.0 (12.8–21.5)	17.9 (13.0–21.8)	0.021
Pre-ECMO MAP^a^ mmHg	57 (40–75)	57 (40–73)	58 (40–79)	0.299
Post-ECMO ABG^a^				
PH	7.40 (7.34–7.46)	7.40 (7.34–7.45)	7.40 (7.34–7.48)	0.071
PaCO_2_, mmHg	36 (31–41)	37 (32–41)	34 (27–41)	< 0.001
PaO_2_, mmHg	150 (92–289)	147 (90–297)	167 (95–271)	0.369
HCO_3_, mmol/L	19.0 (23.0–26.0)	19.6 (23.0–26.0)	18.1 (21.2–24.7)	< 0.001
ΔPaCO2, mmHg	−12 (−31 to 0)	−12 (−31 to 0)	−11 (−31 to 1)	0.747
Rel∆CO2, %	−26 (−48 to 0)	−26 (−47 to 0)	−27 (−51 to 2)	0.370
PaO2 > 300 mmHg	658 (24)	537 (24)	121 (21)	0.092
ECMO characteristics^a^				
Bilateral femoral cannula	1192 (33)	1072 (39)	120 (15)	< 0.001
Distal limb cannula	945 (26)	810 (29)	135 (16)	< 0.001
Cannulation in ER	460 (25)	328 (23)	132 (29)	0.018
Cannulation in OR	160 (9)	97 (7)	63 (17)	< 0.001
Cannulation in ICU	521 (28)	365 (26)	156 (34)	0.001
Cannulation in CCL	615 (33)	535 (38)	80 (17)	< 0.001
VA-ECMO duration (days)	3.0 (1.2–6.1)	3.1 (1.2–6.1)	3.0 (1.0–6.1)	0.294

Data are presented as medians (25th–75th percentile) or n (%)

*IQR* interquartile range, *BMI* body mass index, *COPD* chronic obstructive pulmonary disease, *OHCA* out-of-hospital cardiac arrest, *PVAD* percutaneous ventricular assist device, *ABG* arterial blood gas, *ΔPaCO2* calculated as (24hPost_ECMO_PaCO2–Pre_ECMO_PaCO2), *Rel∆CO2* calculated as ΔPaCO2/Pre_ECMO_PaCO2, *ER* emergency room, *OR* operating room, *ICU* intensive care unit, *CCL* cardiac cath lab, *VA-ECMO* veno-arterial extracorporeal membrane oxygenation

^a^Missing values for some variables resulted in different denominators for the following variables: sex(*n* = 3551), weight(*n* = 3445), BMI(*n* = 1999), comorbid conditions (*n* = 3412), cardiac origin (*n* = 3412), OHCA (*n* = 2017), witnessed (*n* = 2035), initial shockable rhythm (*n* = 1983),pre-ECMO support(*n* = 2933) PH(*n* = 2420), CO_2_(*n* = 2351), PO_2_(*n* = 2321), HCO_3_(*n* = 2279), pre-ECMO MAP (*n* = 1687), post-ECMO ABG(*n* = 2766), Cannulation in ER (*n* = 1872), Cannulation in OR(*n* = 1872), Cannulation in ICU(*n* = 1872), VA-ECMO duration (*n* = 3573)

### Primary outcome

**Fig. 3:**
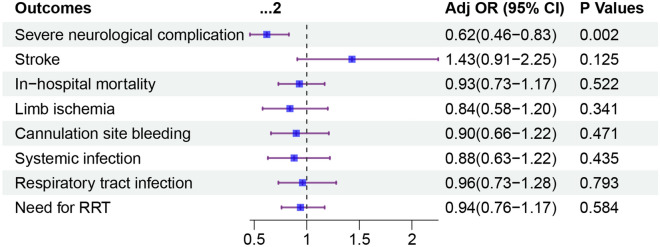
Association of percutaneous cannulation and outcomes in extracorporeal cardiopulmonary resuscitation patients. Data were adjusted for age, weight, sex, race, comorbid conditions, cardiac origin, bilateral femoral cannula, distal limb cannula use, pre-ECMO PH, VA-ECMO duration

**Fig. 4:**
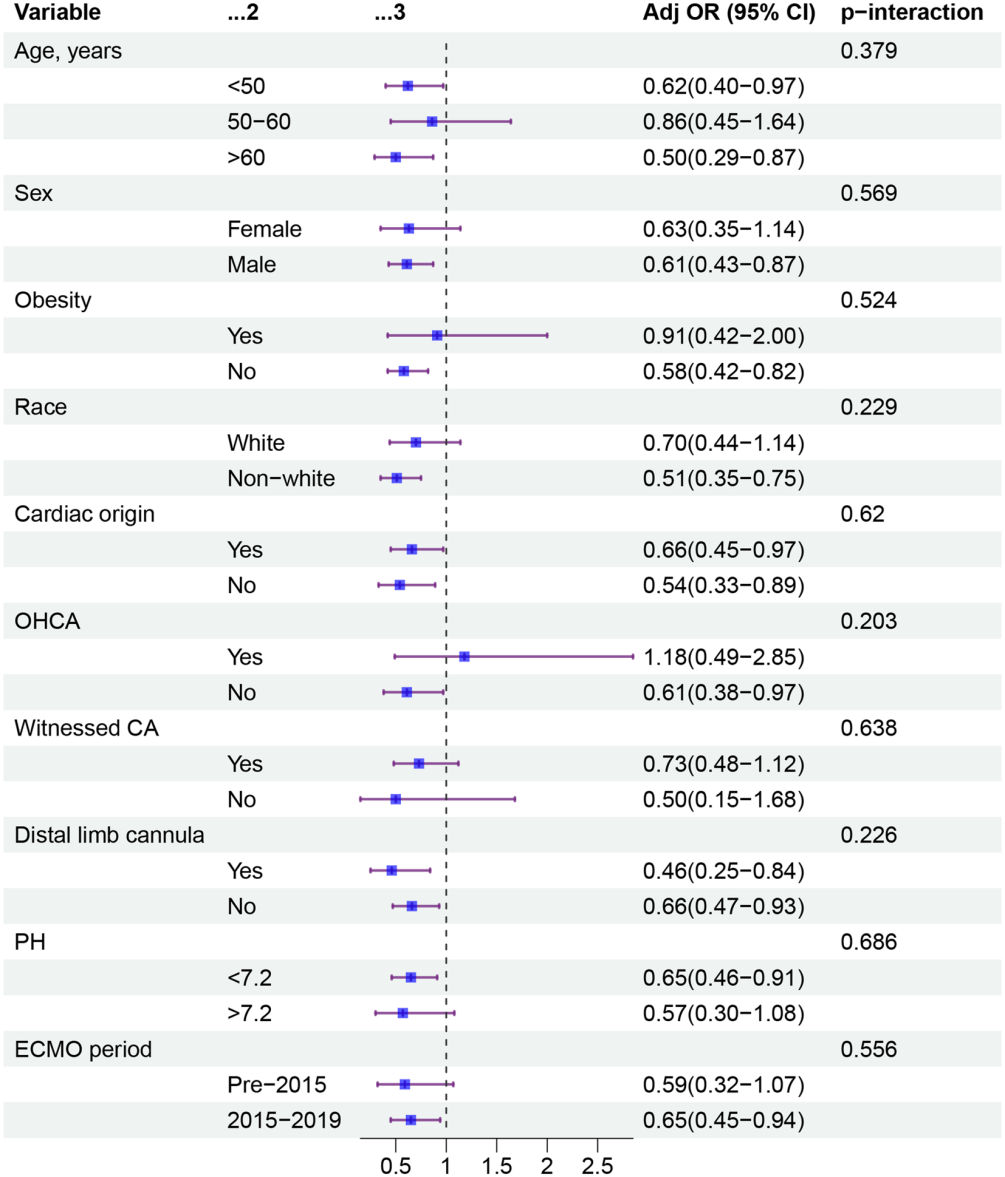
Association between percutaneous cannulation and severe neurological complication across prespecified subgroups. *OHCA* out-of-hospital cardiac arrest, *ECMO* extracorporeal membrane oxygenation

The overall rate of severe neurological complication was 15%. Compared to patients undergoing surgical cannulation, patients undergoing percutaneous cannulation had lower unadjusted rate of severe neurological complication (13% vs 19%; p < 0.001; Table 2). After multivariable logistic regression modeling, percutaneous cannulation was independently associated with a lower risk of severe neurological complication (OR 0.62; 95% CI 0.46–0.83; *p* = 0.002; Fig. 3). This association remained significant when the multivariable models were constructed with omitting pH (Additional file 1: Table S2), adding pre-ECMO support (Additional file 1: Table S3), adding the year of ECPR (Additional file 1: Table S4), or entering oxygenation variables (Additional file 1: Table S5). The association of percutaneous cannulation with lower rate of severe neurological complication remained consistent across most of evaluated subgroups (Fig. 4). There were no significant interactions between percutaneous cannulation and the variables that defined the subgroups.

**Table 2:** Outcomes

Outcome	Overall (*n* = 3575)	Percutaneous group (*n* = 2749)	Surgical group (*n* = 826)	*p* value
Severe neurological complication	524 (15)	370 (13)	154 (19)	< 0.001
Brain death	429 (12)	298 (11)	131 (16)	< 0.001
CNS diffuse ischemia	120 (3)	94 (3)	26 (3)	0.704
Stroke	245 (7)	201 (7)	44 (5)	0.048
In-hospital mortality	2634 (74)	2038 (74)	596 (72)	0.257
Limb ischemia	315 (9)	230 (8)	85 (10)	0.092
Cannulation site bleeding	513 (14)	402 (15)	111 (13)	0.394
Systemic infection	360 (10)	259 (9)	101 (12)	0.019
Respiratory tract infection	603 (17)	460 (17)	143 (17)	0.697
Need for RRT	1280 (36)	960 (35)	320 (39)	0.045

*CNS* central nervous system, *RRT* renal replacement therapy

### Secondary outcomes

The overall in-hospital mortality was 74%. In-hospital mortality in the percutaneous group was similar with that in the surgical group (74% vs 72%; *p* = 0.257; Table 2). In multivariable logistic regression analyses, with adjustment for potential confounders, percutaneous cannulation was not independently associated with in-hospital mortality (OR 0.93; 95% CI 0.73–1.17; *p* = 0.522; Fig. 3). This association remained insignificant in sensitivity analyses (Additional file 1: Tables S2, S3 and S4). The association between percutaneous cannulation and in-hospital mortality remained fairly consistent across clinical subgroups (Additional file 1: Figure S1).

Complications, including stroke, limb ischemia, cannulation site bleeding, systemic infection, respiratory tract infection, and renal failure requiring RRT, were common in both the percutaneous group and the surgical group. In multivariable logistic regression modeling, compared with surgical cannulation, percutaneous cannulation was associated with similar rates of stroke (OR 1.43; 95% CI 0.91–2.25; *p* = 0.125), limb ischemia (OR 0.84; 95% CI 0.58–1.20; *p* = 0.341), cannulation site bleeding (OR 0.90; 95% CI 0.66–1.22; *p* = 0.471), systemic infection (OR 0.88; 95% CI 0.63–1.22; *p* = 0.435), respiratory tract infection (OR 0.96; 95% CI 0.73–1.28; *p* = 0.793), and renal failure requiring RRT (OR 0.94; 95% CI 0.76–1.17; *p* = 0.584; Fig. 3). These findings remained similar in sensitivity analyses (Additional file 1: Tables S2, S3 and S4). Associations between percutaneous cannulation and limb ischemia and cannulation site bleeding in prespecified subgroups are reported in Additional file 1: Figures S2 and S3, respectively.

### Center percutaneous experience and ECPR volume

The median (IQR) volume of center percutaneous ECPR was 4(1–10) cases. Crude rate of severe neurological complication was similar across different levels of center percutaneous experience (< 5 cases, 18%; ≥ 5 cases, 14%; *p* = 0.052). In multivariable modeling, the association of percutaneous cannulation with lower rate of severe neurological complication was observed in both levels of center percutaneous experience (Additional file 1: Table S6), and there was no significant interaction between percutaneous cannulation and center percutaneous experience. Crude rate of severe neurological complication was similar across different levels of center ECPR volume (< 30 cases, 14%; ≥ 30 cases, 15%; *p* = 0.736). In multivariable modeling, the association of percutaneous cannulation with lower rate of severe neurological complication was only observed in centers with < 30 cases of ECPR (Additional file 1: Table S7), and there was no significant interaction between percutaneous cannulation and center ECPR volume. In centers with ≥ 5 cases of percutaneous ECPR or ≥ 30 cases of ECPR, percutaneous cannulation was also associated with a lower risk of limb ischemia (Additional file 1: Tables S6 and S7).

## Discussion

To our knowledge, we report the largest, international, multicenter study comparing percutaneous cannulation to surgical cannulation in adults supported with femoro-femoral ECPR for CA. The main findings of our study are the following: (1) the proportion of patients undergoing percutaneous cannulation has increased exponentially over the past decade and has been higher than that of patients undergoing surgical cannulation since 2012. (2) Percutaneous cannulation was associated with a lower rate of severe neurological complication as compared to surgical cannulation. Older patients (age > 60 years) may benefit most from percutaneous cannulation. (3) Percutaneous cannulation was associated with similar rates of in-hospital mortality and other major complications. (4) The association of percutaneous cannulation and a lower risk of severe neurological complication was consistent across different levels of center percutaneous experience.

### Percutaneous canulation use over time

Despite promising outcomes in patients with CA, the deployment of ECPR is time-sensitive, complex, and labor intensive, resulting in wide variability in cannulation timing, location, and technique [16]. Optimal cannulation technique is essential for ECPR initiation and outcome optimization. Percutaneous cannulation has been shown to be effectively performed by operators from many disciplines. In our study, approximately three-quarter of ECPR patients underwent percutaneous cannulation, and we found a substantial increase in the use of percutaneous cannulation over the past decade, consistent with reports from other observational studies [25, 27]. Advances in cannula designs and ultrasound machines has enabled the percutaneous technique to become more widespread in ECPR [28]. However, clinicians performing this technically challenging procedure must have the requisite training to develop the required skills, and sufficient volume of experience to maintain competency. The advantages of the percutaneous approach include the improved timeliness and the potentially decreased risks of bleeding and infection due to the snug fit of skin and subcutaneous tissue around the cannula. The major disadvantages are the inability to visually determine the appropriate size cannula and the potential for displacement of the needle or guidewire resulting in vessel injury or perforation. The advantages of the surgical approach include the direct visualization of the anatomy and the ability to properly size the cannula and ensure intraluminal placement. The disadvantages include the increased risk for surgical site bleeding and the need for surgical repair for decannulation. Despite the increasing use of the percutaneous approach, the availability of surgical support remains an important component in case of failed percutaneous access attempt.

### Characteristics of patients undergoing percutaneous cannulation

Compared with surgical cannulation, percutaneous cannulation was more frequently used in patients with older age, with obesity, and with lower PH. This indicates that physicians prefer this approach in high-risk and sicker populations, potentially owing to the ready availability and minimal invasiveness of percutaneous cannulation at bedside [17]. In addition, percutaneous cannulation was less frequently used in Asian patients but more frequently used in White and Black patients, which could be explained by different levels of percutaneous experience across the included centers.

### Percutaneous cannulation and severe neurological complication

Although recent data suggest a temporal trend toward improved survival rates in patients receiving ECPR, development of neurologic deficits is common and rate of survival with favorable neurologic outcome remains low [29, 30]. In the absence of studies evaluating the effect of percutaneous cannulation on neurologic outcome in ECPR, the most important finding of our study is the strong association of percutaneous cannulation with a lower rate of severe neurological complication in adult ECPR patients. This association might be explained by the potentially beneficial effect of percutaneous cannulation in CA patients treated with ECPR. With the appropriate use of ultrasound guidance, percutaneous canulation could be performed with a high rate of success and a reduced cannulation time, and a very short median VA-ECMO initiation time of 6 min was reported in a recent percutaneous ECPR study [31]; and low‑flow duration was subsequently shortened, which might be linked to better outcomes in patients undergoing ECPR [32, 33]. Unfortunately, we lack data on cannulation time or low‑flow time during ECPR to elucidate the mechanisms leading to improved neurologic outcome with percutaneous cannulation. Unexpectedly, stroke seemed to be more frequent in the percutaneous group as compared to the surgical group. After controlling for potential confounders, the association between percutaneous cannulation and stroke was insignificant. The association between percutaneous cannulation and a lower risk of stroke not observed in our study, likely due to unavailable imaging examinations or reporting bias.

The association of percutaneous cannulation with a lower risk of severe neurological complication consistent across most of evaluated subgroups. These included patients aged < 50 years vs patients aged 50–60 years vs patients aged > 60 years. We observed that patients aged > 60 years had substantially lower rates of severe neurological complication with percutaneous cannulation. Previous animal studies have indicated that old and young animals present different degrees of neuroinflammation and apoptosis reaction after brain ischemic damage [34]; old animals appear to have a reduced ability for brain cell survival [35]. Similarly, older patients might be at high risk of ischemic brain injury following ECPR and might be more likely to benefit from percutaneous cannulation owing to reduced low-flow times [33]. Although old age was associated with poor neurologic outcomes and hospital mortality, our finding suggested that percutaneous femoral ECPR might be a suitable treatment for older patients with refractory CA.

### Percutaneous cannulation and mortality

In-hospital mortality in the overall study cohort was 74%, which was consistent with reports from other studies (66–76%). [1, 9] Since only 21% of the patients treated with ECPR for refractory OHCA, our results predominantly reflected discharge survival of IHCA cases. Compared with surgical cannulation, percutaneous cannulation was not associated with lower in-hospital mortality in the unadjusted as well as in the adjusted analyses, which differed from that in a recent propensity score-matched study by Danial et al. [24]. Although percutaneous cannulation significantly reduced the rate of neurological complication, it seemed not to translate into mortality reduction. Reasons for this observation will remain speculative. Our results could be explained by a variety of patient and center-specific factors, including reversibility of underlying aetiology, center ECPR experience, and quality of post-resuscitation care. However, these data were unavailable in the ELSO registry. Prospective studies are still needed to determine the effect of percutaneous cannulation on mortality in ECPR patients.

### Percutaneous cannulation and ECMO complications

Despites advances in ECMO techniques, limb ischemia remained a common and serious complication of femoral arterial cannulation [36]. The rate of limb ischemia was low and comparable to those reported in earlier studies [24, 37]. The absence of increased risk of limb ischemia in percutaneous femoral ECPR wan another major finding in our study. This association between percutaneous cannulation and limb ischemia might be explained by systematic use of doppler ultrasound, optimal selection of cannula size, and prophylactic use of distal limb cannula [36, 38, 39]. Importantly, early recognition and prompt treatment usually results in favorable limb and patient outcomes. However, there is no standard of care regarding limb ischemia monitoring. Doppler ultrasound and near-infrared spectroscopy have been proposed to detect limb ischemia in this setting [36, 40]. Patients at high risk of limb ischemia (i.e., females, younger patients, patients with peripheral vascular disease, patients receiving high dose vasopressors) may particularly benefit from these tools.

We also did not observe any difference in the rate of cannulation site bleeding, which was consistent with the findings of the propensity score-matched study by Danial et al. [24]. Minimal invasiveness and appropriate use of ultrasound guidance might have decreased the rate of cannulation site bleeding [41]. In addition, percutaneous cannulation was associated with similar rates of systemic infection, respiratory tract infection, and renal failure requiring RRT, confirming the safety of percutaneous cannulation use in ECPR.

### Center percutaneous experience

There was considerable variability in the center percutaneous experience across the included centers, ranging from 0 to 141 cases. After accounting for center percutaneous experience, percutaneous cannulation was also independently associated with lower rate of severe neurological complication as compared to surgical cannulation. However, the association of percutaneous cannulation with lower rate of severe neurological complication was not observed in high-volume centers. This finding was not surprising given that clinicians in high volume centers might be also experienced in surgical femoro-femoral ECPR. Unexpectedly, percutaneous cannulation was associated with a significantly decreased incidence of limb ischemia among centers with comparatively high level of percutaneous experience (≥ 5 cases) and had an interaction with center percutaneous experience, potentially suggesting centers that more frequently performed percutaneous cannulation might have better outcomes with this approach.

### Study limitations

Our study’s strengths include the use of a large, international cohort of patients with CA and adjusted analyses to control for confounders in the evaluation of patients’ outcomes. However, this study has a number of important limitations. First, given its observational design, there is both residual and unmeasured confounding. For instance, data on low-flow time, cerebral performance category data, survivors’ neurological status, arterial cannula size, cannulation time, pre-ECMO lactate, and anticoagulation parameters were unavailable in the ELSO registry, which might have affected the results. Second, this population was mostly composed of IHCA cases. The usefulness of percutaneous cannulation for OHCA patients might have, therefore, been underestimated. Third, missing values for pre-ECMO pH and pre-ECMO support might limit the internal validity of our study. We attempted to mitigate this issue by repeating all logistic regression analyses by either omitting pH or including pre-ECMO support, and our results remined robust in these sensitivity analyses. Fourth, we were limited to analyzing complications collected in the ELSO registry, there were no data on vessel perforation or arterial dissection at canulation, failure of percutaneous cannulation, cannulation site infection, or vascular complications after decannulation, such as surgical revision for persistent bleeding and lower limb sensory-motor deficit. Finally, only in-hospital outcomes were available, precluding an analysis of longer term survival or functional outcomes. Future research should be directed toward determining the long-term benefits of percutaneous cannulation in adult ECPR patients.

## Conclusions

In this large, multicenter, international registry of CA patients who received percutaneous or surgical cannulation for ECPR, percutaneous cannulation was associated with probable lower rate of severe neurological complication, and similar rates of in-hospital mortality, limb ischemia and cannulation site bleeding. Although the trend is in favor of the use of percutaneous cannulation for ECPR, clinicians should select the appropriate cannulation approach based on their experience.

### Supplementary Information

**Additional file 1: Table S1.** ELSO Registry Data Definitions of ECMO complications. **Table S2.** Sensitivity analyses for the association of percutaneous cannulation with outcomes removing pre-ECMO PH. **Table S3.** Sensitivity analyses for the association of percutaneous cannulation with outcomes adding pre-ECMO support. **Table S4.** Sensitivity analyses for the association of percutaneous cannulation with outcomes adding the year of ECPR. **Table S5.** Sensitivity Analyses for the association of percutaneous cannulation with severe neurological complications adding oxygenation variables. **Table S6.** Multivariable logistic regression model of percutaneous cannulation and outcomes stratified by center experience of percutaneous cannulation. **Table S7.** Multivariable logistic regression model of percutaneous cannulation and outcomes stratified by center ECPR volume. **Figure S1. **Association between percutaneous cannulation and in-hospital mortality across prespecified subgroups. **Figure S2. **Association between percutaneous cannulation and limb ischemia across prespecified subgroups. **Figure S3. **Association between percutaneous cannulation and cannulation site bleeding across prespecified subgroups.

## Data Availability

The data sets used and/or analyzed during the current study are available from the corresponding author on reasonable request.

## Abbreviations

ECPR: Extracorporeal cardiopulmonary resuscitation
VA-ECMO: Veno-arterial extracorporeal membrane oxygenation
CA: Cardiac arrest
IHCA: In-hospital cardiac arrest
OHCA: Out-of-hospital cardiac arrest
ELSO: Extracorporeal Life Support Organization
ICD: International Classification of Diseases
BMI: Body mass index
ABG: Arterial blood gas
RRT: Renal replacement therapy
IABP: Intra-aortic balloon pump
PVAD: Percutaneous ventricular assist device
CNS: Central nervous system
IQR: Interquartile range
NC: Neurological complication
