# Supplementary material for: Percutaneous cannulation is associated with lower rate of severe neurological complication in femoro-femoral ECPR: results from the Extracorporeal Life Support Organization Registry
Source: Ann Intensive Care. 2023 Aug 30;13:77. doi: 10.1186/s13613-023-01174-1 (PMC10469150; doi:10.1186/s13613-023-01174-1)

**Additional Material**

**Additional Table 1. ELSO Registry Data Definitions of ECMO complications**

| **Event** | **Definitions** |
| --- | --- |
| Brain death | The Canadian Neocritical Care Guideline defined brain death as the irreversible loss of the capacity for consciousness combined with the irreversible loss of all brainstem functions, including the capacity to breathe. Neurological determination of death (NDD) is the process and procedure for determining brain death. |
| Central nervous system (CNS) diffuse ischemia | CNS ischemia with CT or MRI demonstrating diffuse ischemic changes. |
| Ischemic stroke | Central nervous system infarction with US, CT, or MRI demonstrating localized ischemic change. |
| Hemorrhagic stroke | Intra/extra parenchymal central nervous system hemorrhage (may be intraparenchymal, subdural, or subarachnoid) or >= Grade 2 intraventricular hemorrhage seen on ultrasound, CT, or MRI. |
| Limb ischemia | Evidence of compartment syndrome, or limb hypoperfusion requiring reperfusion cannula > 6 hours post cannulation, fasciotomy or limb amputation, secondary to ECMO cannulation |
| Cannulation site bleeding | Bleeding from a peripheral cannulation site such as the neck, groin, or axilla. Requiring PRBC transfusion (>20 mL/kg/24 hrs of PRBCs or > 3U PRBCs/24 hrs in adults) and/or surgical intervention (including intravascular hemostatic agent deployment). A reperfusion cannula is a type of peripheral cannulation site. |
| Need for RRT | Necessity of peritoneal dialysis (PD), continuous venovenous hemodiafiltration (CVVHD), continuous venovenous hemofiltration (CVVHF), or hemodialysis, based on the patient’s ultimate mode of therapy |

**Additional Table 2. Sensitivity analyses for the association of percutaneous cannulation with outcomes removing pre-ECMO PH**

| **Outcome** | **Adj OR** | **95% CI** | **p value** |
| --- | --- | --- | --- |
| Severe neurological complication | 0.78 | 0.60-0.99 | 0.045 |
| Stroke | 1.33 | 0.86-2.07 | 0.203 |
| In-hospital mortality | 0.99 | 0.81-1.22 | 0.995 |
| Limb ischemia | 0.78 | 0.57-1.06 | 0.110 |
| Cannulation site bleeding | 0.88 | 0.68-1.14 | 0.341 |
| Systemic infection | 0.95 | 0.72-1.27 | 0.746 |
| Respiratory tract infection | 1.07 | 0.84-1.37 | 0.565 |
| Need for RRT | 0.95 | 0.78-1.14 | 0.560 |

ECMO, extracorporeal membrane oxygenation; RRT, renal replacement therapy.

**Additional Table 3. Sensitivity analyses for the association of percutaneous cannulation with outcomes adding pre-ECMO support**

| **Outcome** | **Adj OR** | **95% CI** | **p value** |
| --- | --- | --- | --- |
| Severe neurological complication | 0.63 | 0.46-0.88 | 0.007 |
| Stroke | 1.36 | 0.83-2.23 | 0.216 |
| In-hospital mortality | 0.94 | 0.72-1.22 | 0.627 |
| Limb ischemia | 0.88 | 0.59-1.30 | 0.513 |
| Cannulation site bleeding | 0.87 | 0.62-1.21 | 0.397 |
| Systemic infection | 0.90 | 0.63-1.29 | 0.565 |
| Respiratory tract infection | 1.24 | 0.90-1.70 | 0.184 |
| Need for RRT | 0.98 | 0.77-1.24 | 0.855 |

ECMO, extracorporeal membrane oxygenation; RRT, renal replacement therapy.

**Additional Table 4. Sensitivity analyses for the association of percutaneous cannulation with outcomes adding the year of ECPR**

| **Outcome** | **Adj OR** | **95% CI** | **p value** |
| --- | --- | --- | --- |
| Severe neurological complication | 0.64 | 0.46-0.88 | 0.006 |
| Stroke | 1.39 | 0.87-2.22 | 0.171 |
| In-hospital mortality | 1.02 | 0.79-1.31 | 0.885 |
| Limb ischemia | 0.66 | 0.46-0.94 | 0.022 |
| Cannulation site bleeding | 0.72 | 0.53-0.99 | 0.041 |
| Systemic infection | 0.78 | 0.56-1.09 | 0.145 |
| Respiratory tract infection | 0.85 | 0.64-1.14 | 0.288 |
| Need for RRT | 1.04 | 0.83-1.30 | 0.758 |

ECMO, extracorporeal membrane oxygenation; RRT, renal replacement therapy.

**Additional Table 5. Sensitivity Analyses for the association of percutaneous cannulation with severe neurological complications adding oxygenation variables.**

|  |  | 95% CI | |  |
| --- | --- | --- | --- | --- |
| Variables | **OR** | **Lower** | **Upper** | **p value** |
| Rel∆CO2 (by 10%) | 0.93 | 0.90 | 0.97 | 0.001 |
| Severe hyperoxemia | 0.84 | 0.59 | 1.21 | 0.352 |
| Percutaneous cannulation | 0.66 | 0.46 | 0.94 | 0.020 |

Age, weight, sex, race, comorbid conditions, cardiac origin, bilateral femoral cannula, distal limb cannula use, pre-ECMO PH, and VA-ECMO duration were entered in the model, but are not shown here. CI, confidence interval; OR, odds ratio. Hosmer–Lemeshow χ^2^, 4.477 with 8 df; p = 0.812.

**Additional Table 6. Multivariable logistic regression model of percutaneous cannulation and outcomes stratified by center experience of percutaneous cannulation.**

| **Outcomes** | **Adj OR** | **95% CI** | **p-interaction** |
| --- | --- | --- | --- |
| Severe neurological complication |  |  | 0.330 |
| No. of PC < 5 | 0.49 | 0.24-0.99 |  |
| No. of PC ≥ 5 | 0.69 | 0.49-0.99 |  |
| Stroke |  |  | 0.005 |
| No. of PC < 5 | 0.46 | 0.11-1.90 |  |
| No. of PC ≥ 5 | 1.95 | 1.13-3.38 |  |
| In-hospital mortality |  |  | 0.682 |
| No. of PC < 5 | 0.87 | 0.47-1.62 |  |
| No. of PC ≥ 5 | 0.97 | 0.74-1.27 |  |
| Limb ischemia |  |  | 0.001 |
| No. of PC < 5 | 5.21 | 1.68-16.14 |  |
| No. of PC ≥ 5 | 0.63 | 0.42-0.95 |  |
| Cannulation site bleeding |  |  | 0.919 |
| No. of PC < 5 | 0.85 | 0.43-1.69 |  |
| No. of PC ≥ 5 | 0.99 | 0.70-1.40 |  |
| Systemic infection |  |  | 0.794 |
| No. of PC < 5 | 1.03 | 0.42-2.51 |  |
| No. of PC ≥ 5 | 0.86 | 0.60-1.25 |  |
| Respiratory tract infection |  |  | 0.318 |
| No. of PC < 5 | 0.62 | 0.30-1.31 |  |
| No. of PC ≥ 5 | 0.98 | 0.71-1.35 |  |
| Need for RRT |  |  | 0.085 |
| No. of PC < 5 | 1.58 | 0.89-2.78 |  |
| No. of PC ≥ 5 | 0.85 | 0.66-1.08 |  |

**Additional Table 7. Multivariable logistic regression model of percutaneous cannulation and outcomes stratified by center ECPR volume.**

| **Outcomes** | **OR** | **95% CI** | **p-interaction** |
| --- | --- | --- | --- |
| Severe neurological complication |  |  | 0.708 |
| No. of ECPR <30 | 0.55 | 0.36-0.85 |  |
| No. of ECPR ≥30 | 0.78 | 0.44-1.39 |  |
| Stroke |  |  | 0.028 |
| No. of ECPR <30 | 0.96 | 0.52-1.77 |  |
| No. of ECPR ≥30 | 3.67 | 1.56-8.64 |  |
| In-hospital mortality |  |  | 0.309 |
| No. of ECPR <30 | 0.74 | 0.51-1.08 |  |
| No. of ECPR ≥30 | 0.81 | 0.53-1.26 |  |
| Limb ischemia |  |  | 0.001 |
| No. of ECPR <30 | 2.13 | 1.07-4.24 |  |
| No. of ECPR ≥30 | 0.48 | 0.25-0.90 |  |
| Cannulation site bleeding |  |  | 0.616 |
| No. of ECPR <30 | 0.91 | 0.60-1.40 |  |
| No. of ECPR ≥30 | 0.86 | 0.50-1.50 |  |
| Systemic infection |  |  | 0.441 |
| No. of ECPR <30 | 1.11 | 0.63-1.96 |  |
| No. of ECPR ≥30 | 0.85 | 0.48-1.50 |  |
| Respiratory tract infection |  |  | 0.316 |
| No. of ECPR <30 | 0.84 | 0.55-1.30 |  |
| No. of ECPR ≥30 | 0.98 | 0.60-1.60 |  |
| Need for RRT |  |  | 0.089 |
| No. of ECPR <30 | 1.19 | 0.85-1.65 |  |
| No. of ECPR ≥30 | 0.68 | 0.46-1.01 |  |

**Additional Figure 1. Association between percutaneous cannulation and in-hospital mortality across prespecified subgroups. OHCA, out-of-hospital cardiac arrest; ECMO, extracorporeal membrane oxygenation.**


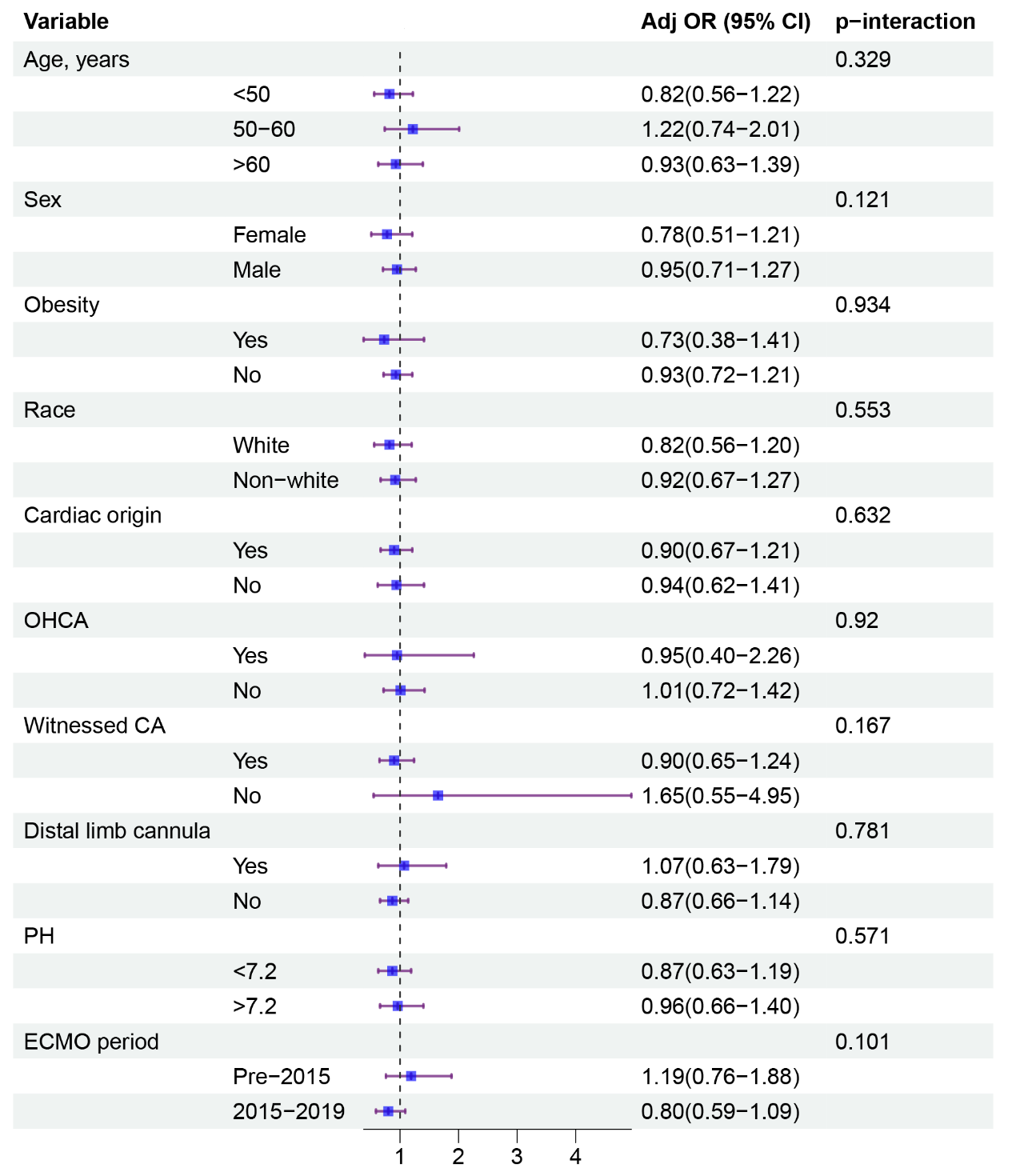


**Additional Figure 2. Association between percutaneous cannulation and** **limb ischemia across prespecified subgroups. OHCA, out-of-hospital cardiac arrest; ECMO, extracorporeal membrane oxygenation.**


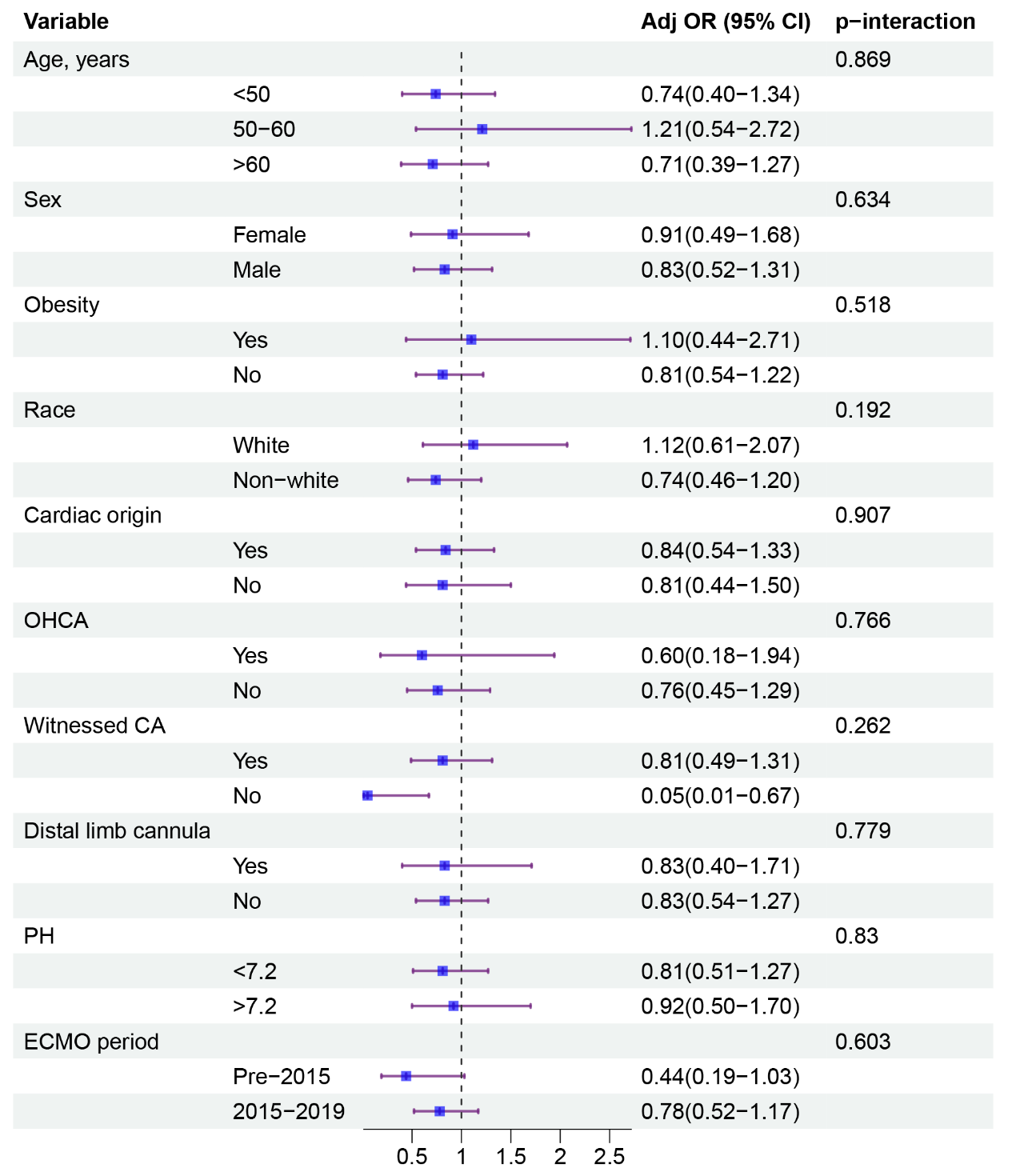


**Additional Figure 3. Association between percutaneous cannulation and** **cannulation site bleeding across prespecified subgroups. OHCA, out-of-hospital cardiac arrest; ECMO, extracorporeal membrane oxygenation.**


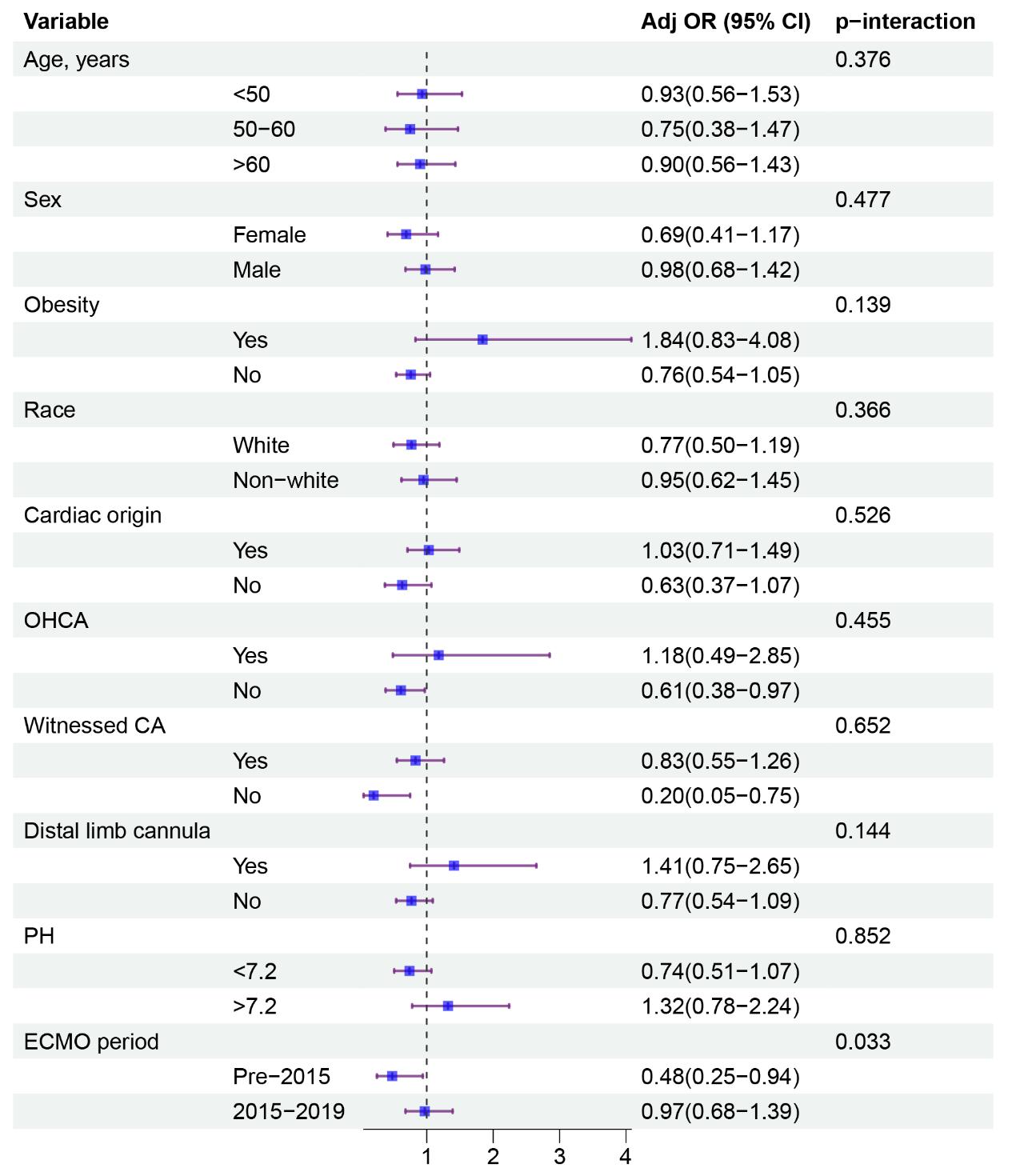

Supplement: Supplementary file 1 — Additional file 1: Table S1. ELSO Registry Data Definitions of ECMO complications. Table S2. Sensitivity analyses for the association of percutaneous cannulation with outcomes removing pre-ECMO PH. Table S3. Sensitivity analyses for the association of percutaneous cannulation with outcomes adding pre-ECMO support. Table S4. Sensitivity analyses for the association of percutaneous cannulation with outcomes adding the year of ECPR. Table S5. Sensitivity Analyses for the association of percutaneous cannulation with severe neurological complications adding oxygenation variables. Table S6. Multivariable logistic regression model of percutaneous cannulation and outcomes stratified by center experience of percutaneous cannulation. Table S7. Multivariable logistic regression model of percutaneous cannulation and outcomes stratified by center ECPR volume. Figure S1. Association between percutaneous cannulation and in-hospital mortality across prespecified subgroups. Figure S2. Association between percutaneous cannulation and limb ischemia across prespecified subgroups. Figure S3. Association between percutaneous cannulation and cannulation site bleeding across prespecified subgroups. [file 13613_2023_1174_MOESM1_ESM.docx]
